# Gene design, cloning and protein-expression methods for high-value targets at the Seattle Structural Genomics Center for Infectious Disease

**DOI:** 10.1107/S1744309111026698

**Published:** 2011-08-13

**Authors:** Amy Raymond, Taryn Haffner, Nathan Ng, Don Lorimer, Bart Staker, Lance Stewart

**Affiliations:** a Seattle Structural Genomics Center for Infectious Disease (SSGCID), USA; b Emerald BioStructures, 7869 NE Day Road West, Bainbridge Island, WA 98122, USA

**Keywords:** structural genomics, community requests, gene engineering, structure-guided construct design, *Gene Composer*, PIPE cloning, LEX bioreactor

## Abstract

Any structural genomics endeavor, particularly ambitious ones such as the NIAID-funded Seattle Structural Genomics Center for Infectious Disease (SSGCID) and Center for Structural Genomics of Infectious Disease (CSGID), face technical challenges at all points of the production pipeline. One salvage strategy employed by SSGCID is combined gene engineering and structure-guided construct design to overcome challenges at the levels of protein expression and protein crystallization. Multiple constructs of each target are cloned in parallel using Polymerase Incomplete Primer Extension cloning and small-scale expressions of these are rapidly analyzed by capillary electrophoresis. Using the methods reported here, which have proven particularly useful for high-value targets, otherwise intractable targets can be resolved.

## Abstract 2

An overview of one salvage strategy for high-value SSGCID targets is given.

## 1. Introduction   

**Figure 1:**
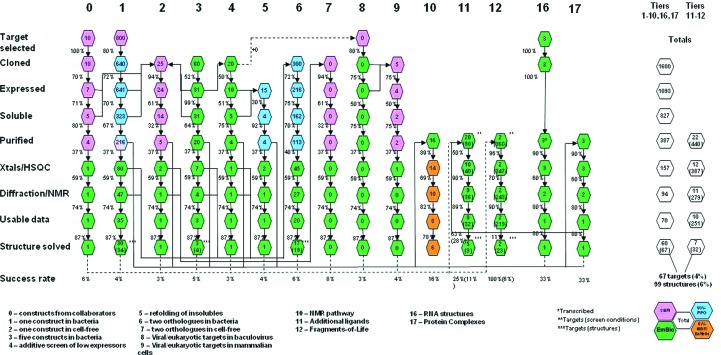
SSGCID multipronged escalating pipeline. Each Tier can be read from top to bottom, with increasing technology applications read from left to right. Annual goal estimates are tabulated on the right, with Tier-specific success rates calculated along the bottom.

The Seattle Structural Genomics Center for Infectious Disease (SSGCID) was established as a collaboration between Seattle Bio­Med, Emerald BioStructures and the University of Washington in 2007. The primary mission of SSGCID is to establish a resource for gene-to-structure research focused on the structure determination of ∼400 protein targets from NIAID Category A–C pathogens, as well as organisms causing emerging and re-emerging infectious diseases (Myler *et al.*, 2009 ▶). To accomplish this, the SSGCID consortium has adapted a multipronged serially escalating approach to protein-structure solution. As shown in Fig. 1 ▶, the SSGCID production pipeline has been outfitted with several technological layers, referred to as ‘Tiers’, which can be applied as salvage strategies. In Tier 1 (upper left corner, progressing from top to bottom), targets are cloned from native sources and protein expression is attempted in a bacterial host. In Tier 2, target genes are subcloned from Tier 1 constructs into the appropriate expression vector for wheat germ cell-free protein expression.

In Tier 3, the native genes are abandoned and all genes are engineered using *Gene Composer* (Raymond *et al.*, 2009 ▶; Lorimer *et al.*, 2009 ▶). Synthetic genes are cloned *via* Polymerase Incomplete Primer Extension (PIPE) cloning (Klock *et al.*, 2008 ▶) into a T7-based protein-expression vector engineered to donate an amino-terminal hexahistidine-Smt fusion and are expressed in bacterial cells. Soluble protein production from each of the six constructs per target is assessed by small-volume protein-expression testing, measuring the amount of recombinant protein partially purified by batch IMAC using magnetic nickel beads (Gaberc-Porekar & Menart, 2001 ▶). All promising con­structs of all targets are then grown as large-scale expression cultures in a LEX bioreactor.

Being a gene-to-structure service for the community at large is the key mission of SSGCID and direct requests from the community are treated as high-value targets. Tier 3 can be utilized as a salvage strategy for any targets that have failed to produce sufficient soluble protein in Tiers 1 and 2. Moreover, Tier 3 also serves as an efficient entry point to the SSGCID pipeline for eukaryotic/viral community request targets or any target for which the requestor has failed to produce soluble protein in the bacterial platform. Here, we describe the gene design, cloning and protein-expression methodologies for high-value SSGCID targets and summarize the utility of these methods in our consortium.

## 2. Materials and methods   

### 2.1. Target gene engineering   

All gene-engineering steps were undertaken using *Gene Composer* software (Lorimer *et al.*, 2009 ▶; Raymond *et al.*, 2009 ▶). The design process began with the full-length target amino-acid sequence, which was backtranslated to allow codon harmonization with the bacterial expression host. Briefly, an *Escherichia coli* codon-utilization table was applied to dictate the frequency with which synonymous codons are used to encode the target protein, with a minimum frequency of 2% required for inclusion. Many additional engineering steps followed, including secondary-structure minimization, G:C content balance, removal of cryptic Shine–Delgarno sequences, addition of second- and third-frame ambush stop codons, relieving extended nucleotide or codon repeats and introduction or removal of restriction sites. All nucleic acid modifications were made without modification of the intended amino-acid sequence.

**Figure 2:**
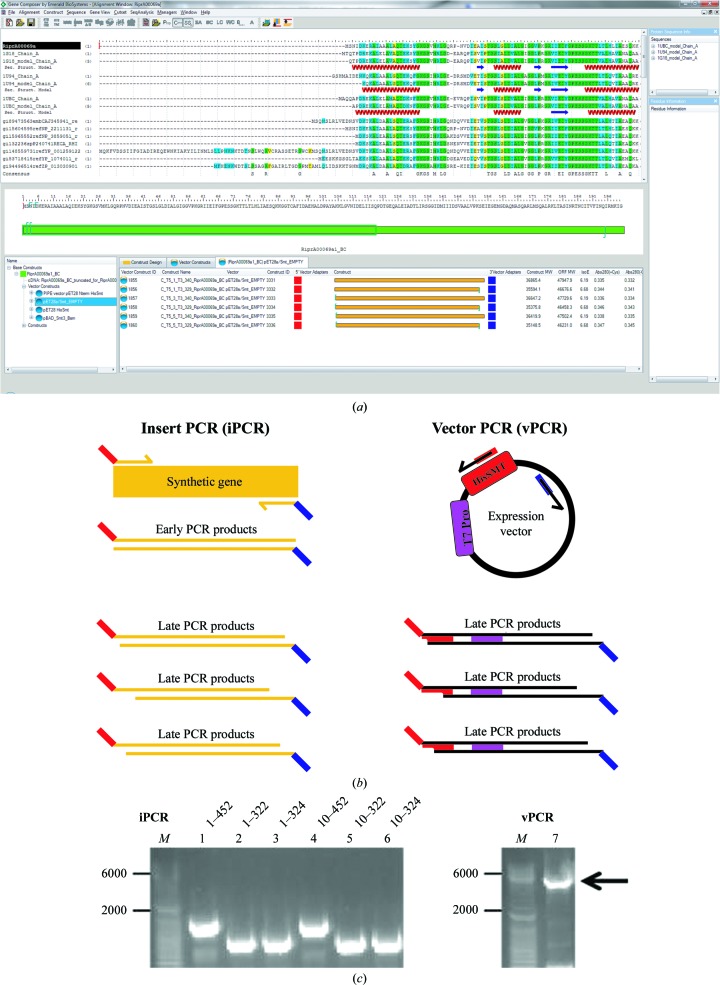
Tier 3 gene design and cloning strategy. (*a*) *Gene Composer* design-session window, showing the target amino-acid base construct in green (middle window) and the structure-guided construct-design products in gold (bottom window). (*b*) Polymerase Incomplete Primer Extension (PIPE) cloning strategy used for this tier of SSGCID pipeline production. Insert PCR products are amplified using primers with homology to the vector termini (shown in red and blue). Vector PCR products are amplified by primers with homology to only the vector termini. (*c*) Agarose-gel analysis of insert PCR (with target amino-acid numbering) and vector PCR products.

Once a nucleic acid sequence had been derived through engineering, alternative protein constructs were designed. The design session aligned the primary structure of the target protein with homologous proteins from the Protein Data Bank (PDB), including all secondary-structure and contact information derived from the PDB files. New protein termini were selected based on conservation of primary structure, secondary structure and structure resolution information. In this way, five alternative constructs were designed with the benefit of all that is known about homologous structures. This strategy is a proven technique to improve crystallization and structure-solution rates (Gräslund *et al.*, 2008 ▶). Example constructs are schematized as gold bars in Fig. 2 ▶(*a*). The engineered gene encoding the full-length protein was purchased from a synthesis vendor and this one synthetic gene served as a template for cloning the full-length and terminal truncation variants.

### 2.2. Cloning   

All clones were produced using PIPE cloning. This is a PCR-based cloning strategy which requires no enzymes beyond the PCR polymerase and allows the cloning of crude PCR products without labor-intensive product purification. In this method, the target gene is amplified in an ‘insert PCR reaction’ by primers with homology to both the gene termini (25-base complementarity) as well as the vector termini (15-base complementarity), while the vector is amplified in a ‘vector PCR reaction’ by primers with only vector complementarity. PIPE cloning is schematized in Fig. 2 ▶(*b*); the insert and vector PCR products are shown in Fig. 2 ▶(*c*). The vector for bacterially expressed targets in this SSGCID Tier was a T7-based expression vector which had been engineered to donate an amino-terminal hexahistidine-Smt tag (MGHHHHHHSGEVKPEVKPETHINLKVSDGSSEIFFKIK­KTTPLRRLMEAFAKRQGKEMDSLRFLYDGIRIQADQTP­EDLDMEDNDIIEAHREQIGG). The Smt tag is very specifically and efficiently removed by UlpI protease, which recognizes the three-dimensional fold of Smt rather than a short primary structure (Mossessova & Lima, 2000 ▶). The digested target protein carries no artifact from the tag, which may be an advantage for crystallographic efforts. In this way, UlpI cleavage serves as a confirmation that the recombinant protein is soluble and properly folded. The PCR cycling excluded the final extension step, allowing the final products to have variably single-stranded termini, which is the necessary result of incomplete primer extension. The crude insert PCR and crude vector PCR reactions were combined in equal volumes and this combination was transformed into chemically competent TOP10 cells. Annealing of the complementary regions on the termini of the insert and vector PCR products created the rare but selectable desired expression plasmid. Two to four colonies were screened by DNA sequencing, generally resulting in an 85% cloning success rate. Failures pre­dominantly occurred at the level of the insert PCR reaction, which can be constrained by the thermodynamics of the terminal nucleotide sequence.

### 2.3. Small-scale expression and expression testing   

Sequence-verified clones were transformed into chemically com­petent BL21 (DE3) cells for protein expression and stored as glycerol stocks at 193 K. Glycerol stocks were streaked on selective agar and freshly grown isolated colonies were used to inoculate 1.2 ml overnight cultures of Terrific Broth (TB) medium supplemented with 0.5% glucose. All small-scale cultures were grown in round-bottom 96-well blocks. This non-inducing culture was grown overnight at 298 K with shaking at 220 rev min^−1^. After approximately 16 h, 40 µl of this overnight culture was used to inoculate 1.2 ml TB medium supplemented with Overnight Express System 1 autoinduction reagents (Novagen). Following inoculation, the 96-well block was allowed to shake at 293 K for approximately 10 min to allow thorough mixing. After mixing, the 1.2 ml culture was split into two 0.6 ml cultures using an additional 96-well block. Small-scale induction cultures were grown for 48 h at 293 K, shaking at 220 rev min^−1^. Cultures were harvested by centrifugation and stored at 253 K for at least 1 h prior to processing.

**Figure 3:**
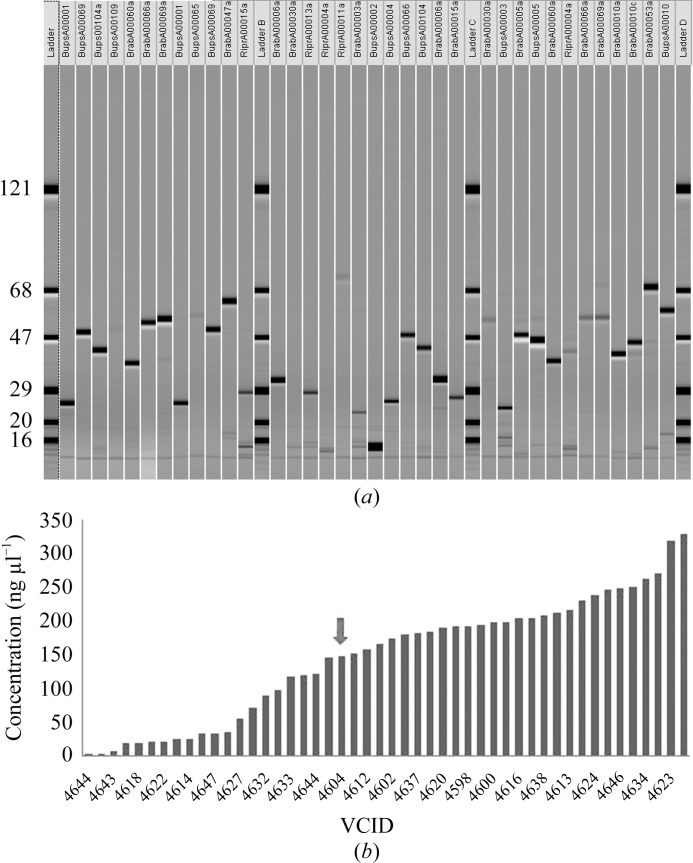
Analysis and quantitation of point-mutant recombinant proteins partially purified on a small scale. (*a*) Virtual gel of capillary electrophoresis by Caliper LapChip 90. Yields vary by mutant. (*b*) Mutant specific protein yields obtained, with wild-type protein indicated by a red arrow.

Frozen bacterial pellets were resuspended and lysed in 50 m*M* NaH_2_PO_4_ pH 8, 300 m*M* NaCl, 10 m*M* imidazole, 1% Tween 20, 2 m*M* MgCl_2_, 1 mg ml^−1^ lysozyme and 0.1 µl ml^−1^ Benzonase and processed essentially as proscribed by the nickel-bead manufacturer (Qiagen). Chemical lysis was allowed to proceed by 30 min of vigorous shaking at room temperature. The crude lysate was clarified by centrifugation for 30 min at 4000 rev min^−1^ and 277 K. The soluble fraction was combined with magnetic Ni–NTA beads in a V-bottom microtiter plate and allowed to react for 1 h with shaking at 289 K. The unbound soluble protein was removed and the magnetic nickel beads were washed twice with 200 µl wash buffer: 50 m*M* NaH_2_PO_4_ pH 8, 300 m*M* NaCl, 20 m*M* imidazole and 0.05% Tween 20. The washed proteins were eluted in 5 min with 60 µl elution buffer: 50 m*M* NaH_2_PO_4_ pH 8, 300 m*M* NaCl, 250 m*M* imidazole and 0.05% Tween 20. A portion of each elution product was reacted with UlpI protease for 30 min at room temperature. Both the untreated and the protease-treated elution products were analyzed by capillary electrophoresis in a LabChip 90 (Caliper), as shown in Fig. 3 ▶. Alternatively, all fractions from the expression testing can be analyzed by SDS–PAGE.

### 2.4. Large-scale expression   

Inoculum cultures of TB medium supplemented with antibiotics (50 µg ml^−1^ kanamycin) were grown for approximately 18 h at 310 K. TB auto-induction medium was freshly prepared according to the manufacturer’s protocol (Novagen) and was supplemented with antibiotics. The bottles were inoculated with 3 ml overnight culture. Inoculated bottles were placed into a LEX bioreactor (Harbinger Biotech, Ontario, Canada). Cultures were grown for approximately 60–72 h at 293 K. To harvest, the culture was centrifuged at 4000*g* for 20 min at 277 K. A 10 ml aliquot of the culture was processed separately and screened for total protein, soluble protein and the fraction that binds to immobilized metal-affinity chromatography (IMAC) to predict which large-scale expressions were worth processing further for purification. Until that time, the cell paste was stored at 193 K.

### 2.5. Protein purification   

All aspects of protein purification are covered in detail in Smith *et al.* (2011 ▶).

## 3. Results and discussion   

The Seattle Structural Genomics Center for Infectious Disease (SSGCID) is committed to and achieving the goal of determining 75–­100 three-dimensional protein structures per year from NIAID Category A–C and emerging/re-emerging infectious disease organisms. SSGCID employs a high-throughput gene-to-structure pipeline involving a multi-pronged serial escalation approach to protein expression in bacterial, wheat germ cell-free translation, baculovirus and mammalian systems followed by structure solution using X-ray crystallography and NMR spectroscopy. Proactive engagement of the infectious disease research and drug-therapy communities in the target-selection process helps to ensure that the resulting protein structures provide a blueprint for structure-based drug design of new therapeutics to combat infectious diseases. Moreover, the SSGICD pipeline serves as a gene-to-structure service for the community at large.

Community-requested targets are considered to be particularly high-value targets. Where appropriate, these targets are processed through Tier 3 of the escalating pipeline, which is the focus of this report. The majority of the structures are solved either as apoproteins or as complexes with native ligand. In some cases, however, high-value targets are pursued as binary ligand complexes as a means to further inform drug-design and discovery efforts. Fragment screening can introduce an additional level of challenge. The target protein must not only form high-resolution crystals which can be solved, but the crystal form must also be amenable to soaking with the compound library. One such case is highlighted in Fig. 3 ▶, in which the target is readily crystallizable but a different crystal form is sought.

**Figure 4:**
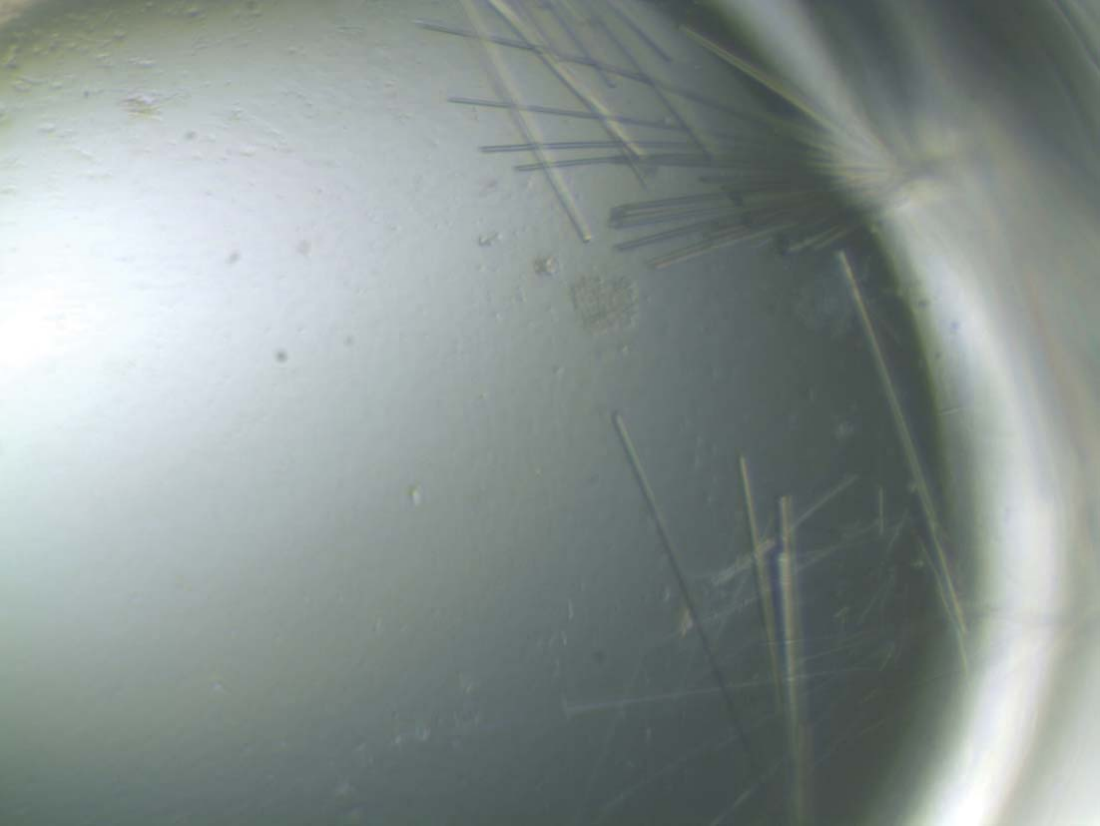
Protein crystal of polymerase PB2 subunit from 2009 pandemic influenza H1N1.

**Figure 5:**
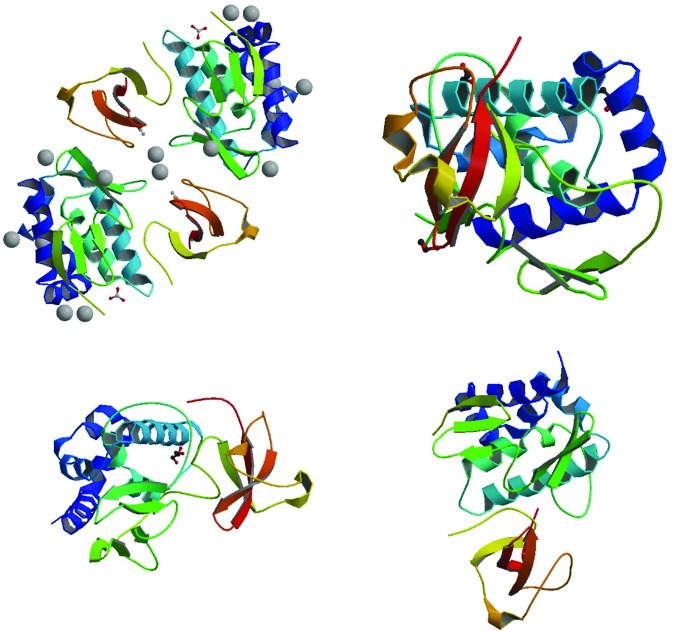
Protein structures of influenza polymerase subunit PB2 from a variety of viral strains obtained using the methods described in this publication. PDB codes (clockwise from top left): 3r2v (T. E. Edwards, A. S. Gardberg & B. Sankaran, unpublished work), 3kc6 (Yamada *et al.*, 2010 ▶), 3l56 (Yamada *et al.*, 2010 ▶) and 3khw (Yamada *et al.*, 2010 ▶).

In addition to our internally selected and community-requested targets, the methods reported here have also enabled a rapid response to emerging diseases such as the 2009 pandemic influenza H1N1 (Yamada *et al.*, 2010 ▶). Example protein crystals of influenza polymerase PB2 subunit are shown in Fig. 4 ▶ and a panel of influenza structures deposited in the Protein Data Bank (PDB) as a result of these methods are highlighted in Fig. 5 ▶. An outcome analysis focused on the SSGCID influenza targets is shown in Table 1 ▶, which reports the success of each target at each step and the overall success rates resulting from the use of these Tier 3 methods. Judicious use of the Tier 3 methodology has enabled efficient production and evaluation of alternative constructs, which in turn accelerates our structure-solution pipeline.

## Figures and Tables

**Table 1:** Outcome analysis for influenza polymerase targets processed by the methods described here from a variety of viral strains Each subunit of the heterotrimeric polymerase from each strain is treated as a separate target (with SSGCID identifier given), for which 27 structure-guided terminal truncation constructs were designed. The structure-determination pipeline is broken down into five distinct steps: cloning, solubility testing, protein purification, protein crystal formation and structure solution. Percentage success overall is calculated by target to account for the multiple constructs designed for each target; percentage success per step is calculated by construct.

Strain	Polymerase subunit	Target	Constructs	Cloned	Soluble	Purified	Crystals	Structure
Avian-1023 H5N1	PB2	InvaA.07055.a	7	7	3	3	2	2
	PB1	InvaA.07056.a	2	2	0	0	0	0
	PA	InvaA.07057.a	6	6	2	2	1	0
Avian-2017 H2N3	PB2	InvaB.07055.c	7	7	2	2	2	1
	PB1	InvaB.07056.c	2	2	0	0	0	0
	PA	InvaB.07057.c	6	6	0	0	0	0
Equine-1 H7N7	PB2	InvaC.07055.b	7	7	3	3	3	0
	PB1	InvaC.07056.b	2	2	1	1	0	0
	PA	InvaC.07057.b	6	6	2	2	0	0
Swine-04 H1N1	PB2	InvaD.07055.a	7	5	3	3	1	1
	PB1	InvaD.07056.a	2	2	0	0	0	0
	PA	InvaD.07056.a	5	5	4	4	1	0
Swine-InDRE4487 H1N1	PB2	InvaE.07055.a	6	6	3	3	1	1
	PA	InvaE.07057.a	5	5	2	2	0	0
Swine-05 H1N1	PA	InvaF.07057.a	4	4	3	3	0	0
Total		15	74	72	28	28	11	5
Overall success (%)				100	73	73	47	27
Success per step (%)				97	39	100	39	45
